# Exosomal S100A4 derived from highly metastatic hepatocellular carcinoma cells promotes metastasis by activating STAT3

**DOI:** 10.1038/s41392-021-00579-3

**Published:** 2021-05-26

**Authors:** Haoting Sun, Chaoqun Wang, Beiyuan Hu, Xiaomei Gao, Tiantian Zou, Qin Luo, Mo Chen, Yan Fu, Yuanyuan Sheng, Kaili Zhang, Yan Zheng, Xudong Ren, Shican Yan, Yan Geng, Luyu Yang, Qiongzhu Dong, Lunxiu Qin

**Affiliations:** 1 Department of General Surgery, Huashan Hospital, Cancer Metastasis Institute, Fudan University, Shanghai, China; 2 Institutes of Biomedical Sciences, Fudan University, Shanghai, China

**Keywords:** Gastrointestinal cancer, Metastasis, Metastasis

## Abstract

Intercellular cross-talk plays important roles in cancer progression and metastasis. Yet how these cancer cells interact with each other is still largely unknown. Exosomes released by tumor cells have been proved to be effective cell-to-cell signal mediators. We explored the functional roles of exosomes in metastasis and the potential prognostic values for hepatocellular carcinoma (HCC). Exosomes were extracted from HCC cells of different metastatic potentials. The metastatic effects of exosomes derived from highly metastatic HCC cells (HMH) were evaluated both in vitro and in vivo. Exosomal proteins were identified with iTRAQ mass spectrum and verified in cell lines, xenograft tumor samples, and functional analyses. Exosomes released by HMH significantly enhanced the in vitro invasion and in vivo metastasis of low metastatic HCC cells (LMH). S100 calcium-binding protein A4 (S100A4) was identified as a functional factor in exosomes derived from HMH. S100A4^rich^ exosomes significantly promoted tumor metastasis both in vitro and in vivo compared with S100A4^low^ exosomes or controls. Moreover, exosomal S100A4 could induce expression of osteopontin (OPN), along with other tumor metastasis/stemness-related genes. Exosomal S100A4 activated OPN transcription via STAT3 phosphorylation. HCC patients with high exosomal S100A4 in plasma also had a poorer prognosis. In conclusion, exosomes from HMH could promote the metastatic potential of LMH, and exosomal S100A4 is a key enhancer for HCC metastasis, activating STAT3 phosphorylation and up-regulating OPN expression. This suggested exosomal S100A4 to be a novel prognostic marker and therapeutic target for HCC metastasis.

## Introduction

Metastasis and recurrence, causing about 90% of deaths of cancer patients, are the most significant characteristics of malignant cancers.^1,2^ Tumor recurrence occurs in over half of hepatocellular carcinoma (HCC) patients at 5 years after resection and is a common cause of poor prognosis.^3^ Intratumor heterogeneity contributes to drug resistance and tumor relapse following therapy.^4^ Understanding phenotypical intratumor heterogeneity of HCC should provide critical knowledge about its diverse metastatic potential.

Intercellular crosstalk plays important roles in cancer progression and metastasis. Classical crosstalk includes cell-to-cell contact and secretion of soluble molecules such as growth factors and cytokines. Exosomes, an efficient intercellular signal delivery system working in both nearby and distant sites, have been identified to play a key role in cancer metastasis in recent years.^5^ These nanovesicles with lipid bilayer membrane contain a variety of contents including proteins, lipids, and nucleic acids, derived from multivesicular bodies.^6^ Exosome-mediated intercellular communication is widely accepted as a powerful promotor to cancer invasiveness.^7^ Al-Nedawik et al. reported that glioma cells with EGFRvIII mutation were highly invasive and could significantly enhance the malignancy of EGFRvIII wild-type cells, via delivering mutated EGFRvIII protein through exosomes.^8^ Exosomes from highly metastatic melanomas could increase the malignancy low metastatic ones by educating bone marrow progenitor cells.^9^ However, the understanding of intercellular communication via exosomes between HCC cells with different metastatic potential is limited.

S100 calcium-binding protein A4 (S100A4) is a member of the S100 family. S100A4 plays an important role in tumor metastasis by regulating adhesion,^10^ extracellular matrix remodeling,^11,12^ and cellular motility.^13,14^ S100A4 secreted from liver cancer-associated-mesenchymal stem cells results in increased HCC invasiveness via miR155-SOCS1-MMP9 axis.^15^ S100A4 could be upregulated in urothelial cells after bladder cancer exosomes treatment.^16^ And recently, S100A4 was reported to regulate pre-metastatic niche in oncogenic pancreatic exosomes.^17^

In this study, we investigated the protein profile of exosomes derived from HCC cells with diverse metastatic potential. Exosomal S100A4 derived from highly metastatic HCC cells (HMH) enhanced the stemness and metastatic potential of low metastatic HCC cells (LMH), indicating that exosomes could mediate interplay within tumor ecosystem, affecting phenotypical intratumor heterogeneity.

## Results

### Isolation and identification of exosomes released and up-taken by HCC cells

**Fig. 1:**
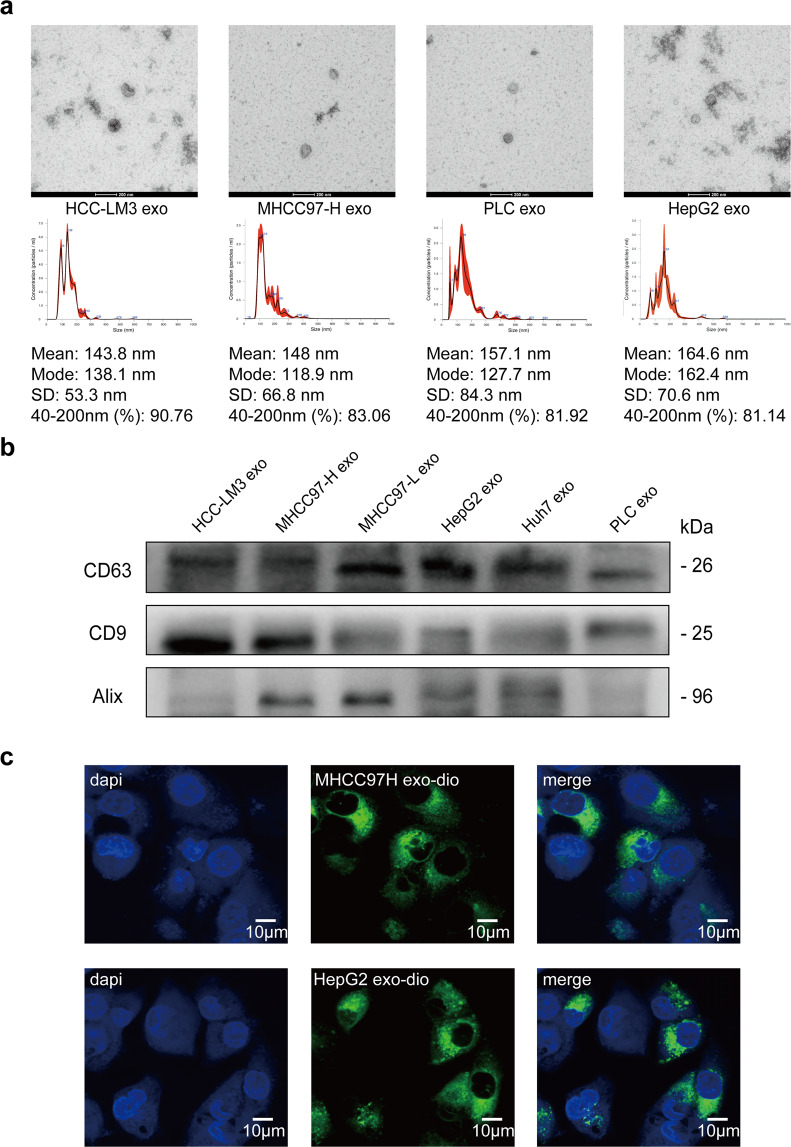
Isolation and identification of exosomes released and up-taken by HCC cells. **a** Transmission electron microscopy of isolated exosomes from HCC cells (HCC-LM3, MHCC97-H, PLC, and HepG2, Scale bar: 200 nm). The concentration and size distribution were determined by nanoparticle tracking analysis (NTA). **b** Western blot of exosomal marker CD63, CD9, and Alix in exosomes isolated from HCC cell lines (HCC-LM3, MHCC97-H, MHCC-97-L, HepG2, Huh7, and PLC). **c** Laser scanning confocal microscope images of DIO treated exosomes (MHCC97H exo and HepG2 exo) up-taken by low metastatic HCC cells (MHCC97-L)

Exosomes were extracted from supernatant of HCC cells by ExoQuick reagent kit. Then morphological observation was performed by transmission electron microscope and shapes of exosomes were observed (Fig. 1a). Furthermore, we utilized nanoparticle tracking analysis (NTA) to determine exosomes diameters, which were mostly ranged between 40 and 200 nm (Fig. 1a). Relatively specific exosomal markers, CD63, CD9, and Alix, were detected by Western blot in six HCC cell lines (Fig. 1b). In order to verify the reliability of extracted exosomes, we adopted methylated-β-Cyclodextrin (MβCD) treatment, which can destruct exosomal lipid membrane. After MβCD treatment, exosomal markers were much less detected by Western blot, which further verified the reliability and purity of exosomes extracted by ExoQuick reagent kit (Supplementary Fig. S1c). Next, we used DIO green to separately dye exosomes released by highly metastatic HCC cells (MHCC97-H, the exosomes were marked as HMH-exosomes) and low metastatic HCC cells (HepG2, the exosomes were marked as LMH-exosomes). We also observed with laser scanning confocal microscope that both green coated HMH-exosomes and LMH-exosomes could be up taken efficiently by lowly metastatic HCC cells, MHCC97-L, and HepG2 (Fig. 1c). These indicate that we have successfully isolated and purified exosomes form HCC cells, and demonstrated that they can be up-taken by the other HCC cells.

### HMH-exosomes enhance the metastatic potential of low metastatic HCC cells

**Fig. 2:**
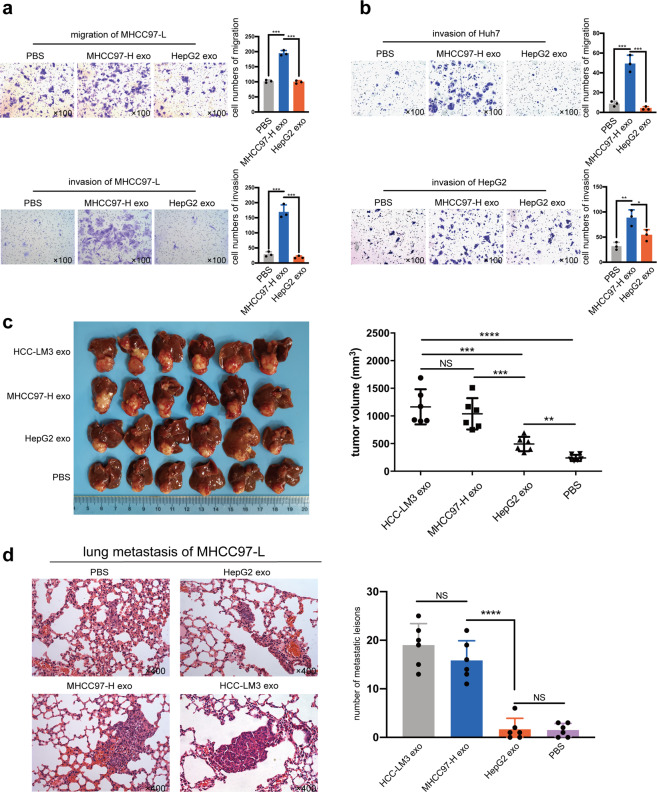
Highly metastatic HCC cells (HMH) derived exosomes enhance metastatic potential of low metastatic HCC cells (LHM) both in vitro and in vivo. **a**, **b** Assessment of migration and invasion in vitro. LHM (MHCC97-L, Huh7, and HepG2) pre-treated with MHCC97-H exosomes, HepG2 exosomes, or PBS (as negative control) for 24 h. The migration ability of MHCC97-L and invasion ability of all the three cell lines were significantly enhanced by MHCC97-H exosomes compared to the control groups. **c** Orthotopic implantation liver tumors from nude mice. MHCC97-L cells treated with HMH exosomes (HCC-LM3 exosomes or MHCC97-H exosomes) formed larger liver tumors. **d** Lung metastasis of mice injected with MHCC97-L. MHCC97-L cells treated with HMH exosomes formed significantly more lung metastatic lesions. All the in vitro assays were conducted three times with three repetitions. Error bars represent the mean ± SD, and the dots represent the value of repetitions in one experiment; *P < 0.05, **P < 0.01, ***P < 0.001, ****P < 0.0001, ns: no significance. An unpaired t test was employed in (**a**) and (**b**), one-way ANOVA followed by Bonferroni’s post hoc test was employed in (**c**) and (**d**)

To investigate the possible role of exosomes in cell-to-cell crosstalk of HCC metastasis, we used HMH-exosomes to pre-treat low metastatic HCC cells (MHCC97-L, Huh7, and HepG2) for 24 h (LMH-exosomes and PBS as control, respectively). After pretreatment, MHCC97-L cells underwent both migration and invasion assays, while Huh7 and HepG2 cells only invasion assays, to test their metastatic potential in vitro. It was noted that significantly more HMH-exosomes pre-treated MHCC97-L cells passed through the Transwell chamber, but not those treated with LMH-exosomes or PBS (Fig. 2a). Similar results were observed in HMH-exosomes pre-treated Huh7 and HepG2 cells, with an exception of LMH-exosomes pre-treated HepG2 cells. They also saw a slightly enhanced invasion, compared to PBS ones (Fig. 2b, Supplementary Fig. S1a, b). These suggest that HMH-exosomes, exosomes released by highly metastatic HCC cells, MHCC97-H, and HCC-LM3, could enhance the in vitro migration and invasion abilities of low metastatic HCC cells.

Then, we established in vivo orthotopic implantation xenograft model with MHCC97-L cells. First, we implanted small tumor tissues into the left liver of nude mice. After one week, PBS, HepG2-exosomes, HCC-LM3-exosomes, and MHCC97-H exosomes were respectively injected into tail veins of these nude mice twice a week for 6 weeks. Then they were euthanized for liver tumor and lung metastasis evaluation. We found that liver tumors of HMH-exosomes groups (HCC-LM3 exosomes group and MHCC97-H exosomes group) were larger than the other two groups (Fig. 2c). Similarly, number of lung metastasis in the HMH-exosomes groups were also significantly larger than the other two groups (Fig. 2d). These demonstrate that HMH-exosomes could significantly enhance in vivo growth and lung metastasis of low metastatic HCC cells.

### Exosomal S100A4 is a key enhancer of metastatic potential in HCC cells

**Fig. 3:**
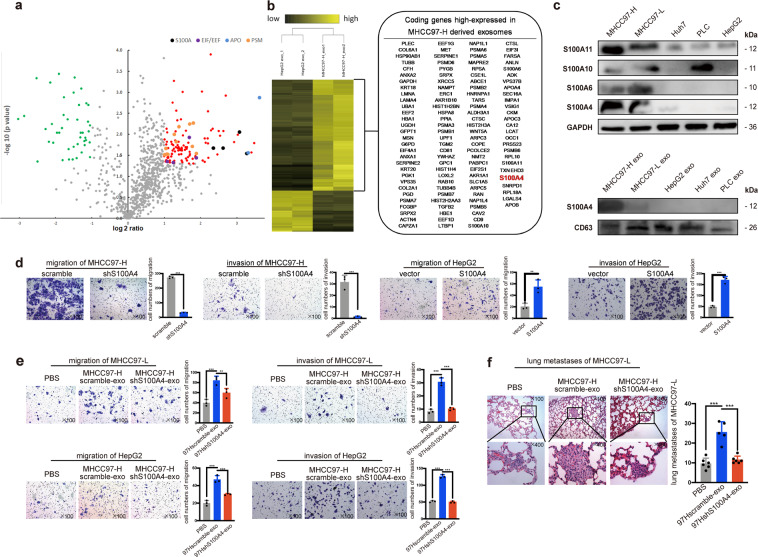
Exosomal S100A4 is a key promoter to enhance metastatic potential. **a** iTRAQ mass spectrum screening was performed to analyze the protein cargo of exosomes derived from MHCC97-H (HMH-exosomes) and HepG2 exosomes (LMH-exosomes). Proteins were clustered at the 2-fold changes with a *p* value less than 0.05. The volcano plots revealed a total 116 up-regulated proteins (right quadrant, generally in red, S100A family in black, EIF/EEF family in purple, APO family in blue and PSM family in orange), and yet 43 proteins were found down-regulated (left quadrant, in green). **b** The results are also expressed as a heat map, from which 116 up-regulated proteins are shown. **c** Diverse expression abundance of S100A protein family in HCC cell lines. Western blot showed that expressions of S100A4 were the highest in MHCC97-H among HCC cell lines. S100A4 was only highly expressed in exosomes derived from MHCC97-H. **d** S100A4 knock-down in MHCC97-H results in inhibited migration and invasion ability. Overexpression of S100A4 enhanced migration and invasion in HepG2. **e** S100A4^rich^ exosomes significantly promotes migration and invasive abilities of cells. Exosomal S100A4 promotes the migration and invasive abilities of cells in vitro. Exosomes of MHCC97-H cells with S100A4 knockdown were defined as S100A4^low^ exosomes, ones derived from the scrambled counterparts were defined as S100A4^rich^ exosomes. S100A4^rich^ exosomes were used to treat MHCC97-L and HepG2 cells for 24 h before migration and invasion assays while PBS and S100A4^low^ exosomes were used as negative controls. **f** Exosomal S100A4 promotes the metastatic potential in vivo. MHCC97-L cells treated with PBS, S100A4^rich^ exosomes or S100A4^low^ exosomes were injected in nude mice to form lung metastasis model. All the in vitro assays were conducted three times with three repetitions. Error bars represent the mean ± SD, and the dots represent the value of each experiment; *P < 0.05, **P < 0.01, ***P < 0.001, ****P < 0.0001, ns: no significance. Statistical significance was determined by unpaired *t* test

In order to identify the element via which HMH-exosomes work to enhance the metastatic potential of low metastatic HCC cells, we adopted iTRAQ mass spectrum screening for HMH-exosomes and LMH-exosomes. Results showed that 116 proteins were significantly up-regulated in HMH-exosomes group while 43 down-regulated (Fig. 3a, b). Proteins were clustered at the 2-fold changes with a *p* value less than 0.05. The volcano plots revealed that 4 protein families, Apo, PSM, EEF/EIF, and S100 calcium binding protein family, were clustered. After reviewing literatures, we focused on four members of S100 calcium binding protein family with the most differentiated expression, S100A4 (means ratio: 9.642), followed sequentially by S100A11 (8.393), S100A10 (6.132), and S100A6 (5.101). Furthermore, we conducted comparison of the expression abundance of S100A4, S100A6, S100A10, and S100A11 in 5 HCC cells lines, as well as the abundance of S100A4 in exosomes derived from these cell lines. Results were consistent with the iTRAQ data (Fig. 3c). Immunohistochemistry (IHC) staining of S100A4 in nude mice tumor models also confirmed that S100A4 was significantly more highly expressed in MHCC97-H derived tumors (Supplementary Fig. S1d). Therefore, we hypothesized that S100A4 may be one of the most potentially functional factors in HMH-exosomes and we selected it for further analyses.

We knocked down S100A4 in HCC-LM3 and MHCC97-H cells, and overexpressed S100A4 in PLC, Huh7, and HepG2 cells (Supplementary Fig. S2a). We then repeated in vitro migration and invasion assays. As a result, after S100A4 was down-regulated, the abilities of migration and invasion of HCC-LM3 and MHCC97-H cells were both inhibited. On the other hand, S100A4 overexpression greatly enhanced Huh7 and HepG2 migration and invasion (Supplementary Fig. S2b). These support that S100A4 is important for in vitro migration and invasion of HCC cells.

Since S100A4 protein could maintain cell stemness, which is important in promoting cancer metastasis, therefore, we performed in vitro sphere formation and in vivo tumor initiation assays. After S100A4 was knocked down, HCC-LM3 and MHCC97-H cells formed much fewer and smaller spheres than the controls (Supplementary Fig. S2c). As expected, when S100A4 was overexpressed in Huh7 and HepG2 cells, they formed significantly more and larger spheres (Supplementary Fig. S2c). In vivo models showed that the tumor initiation time of S100A4 down-regulated HCC-LM3 and MHCC97-H cells was sharply delayed for 1 to 2 weeks depending on the number of tumor cells implanted, and tumor sizes were also smaller as expected (Supplementary Fig. S3). These provide further support that S100A4 could enhance the stemness of HCC cells.

We then further investigated the function of exosomal S100A4 protein on metastatic potential and stemness enhancement. Firstly, we extracted exosomes from supernatants of S100A4 knock-down HCC-LM3 and MHCC97-H cells, and verified that S100A4 expression in those exosomes was significantly lower than the controls (Supplementary Fig. S4). We defined them as S100A4^low^ and S100A4^rich^ exosomes. S100A4^rich^ exosomes were used to pre-treat low metastatic HCC cells, MHCC97-L and HepG2, for 24 h, controlled by S100A4^low^ exosomes and PBS. After pre-treatment, MHCC97-L and HepG2 cells were harvested for migration and invasion assays, to test their metastatic potential in vitro. We observed significantly more S100A4^rich^ exosomes pre-treated MHCC97-L and HepG2 cells passed through the Transwell chamber than controls (Fig. 3e). Then, we conducted in vivo experiment with tail vein injection lung-metastasis model of MHCC97-L cells. First, we pre-treated MHCC97-L cells by PBS, S100A4^rich^ exosomes or S100A4^low^ exosomes for 24 h. Then 50,000 of these pre-treated cells, with their corresponding exosomes or PBS, were injected into tail veins of nude mice. After that, exosomes or PBS was injected into tail vein twice a week. Four weeks later, mice were euthanized and their lungs harvested for paraffin fixation. Results showed that lung metastasis was significantly higher in S100A4^rich^ exosomes treated group than the other two groups (Fig. 3f). In addition to metastatic promotion, in vitro sphere formation rates of MHCC97-L and HepG2 cells were also elevated by S100A4^rich^ exosomes treatment (Supplementary Fig. S5a), so was the in vivo tumor initiation ability of MHCC97-L (Supplementary Fig. S5b). Taken together, these findings further demonstrate the functional roles of exosomal S100A4 in enhancement of the metastatic potential and stemness of HCC cells.

### Exosomal S100A4 activates OPN transcription via STAT3 phosphorylation

**Fig. 4:**
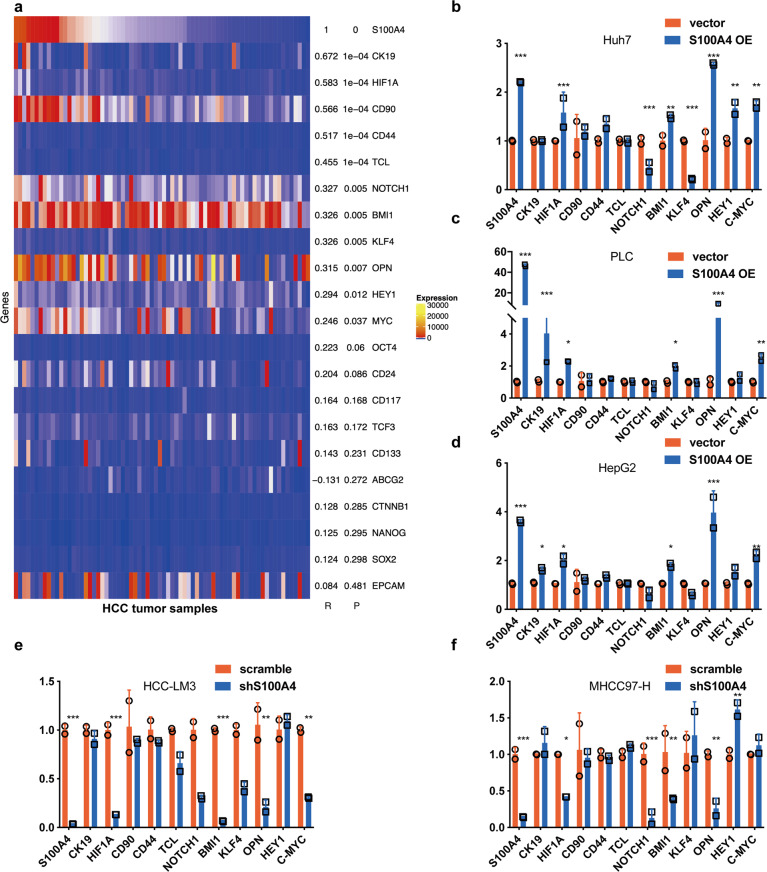
S100A4 expression positively correlates with OPN. **a** Correlation between S100A4 and stemness-related genes. The GEO datasheet GSE39791 were used for correlation analyzes. Among the selected genes, 11 genes including OPN showed significant positive correlation with S100A4. Real-time PCR analyzes revealed the change of mRNA expression after S100A4 overexpression in low metastatic HCC cell lines, Huh7 (**b**), PLC (**c**), and HepG2 (**d**). In highly metastatic HCC cell lines, HCC-LM3 (**d**) and MHCC97-H (**f**), with S100A4 knockdown. Only OPN and its downstream genes (HIF1 and BMI1) in accordance with the tendency of S100A4 expression after knockdown and overexpression. Real-time PCR was conducted three times with two repetitions, and the dots represent the value of repetitions in one experiment. Error bars represent the mean ± SD; *P < 0.05, **P < 0.01, ***P < 0.001, ****P < 0.0001

Next, we attempted to elucidate the mechanism of exosomal S100A4 on metastatic potential and stemness. First, we selected 21 cancer stemness-related genes, OPN, OCT4, NANOG, SOX2, HIF1α, BMI1, ABCG2, CK19, NOTCH1, KLF4, CD44, CD90, CD133, CD117, CD24, EPCAM, TCF3, TCL, CTNNB1, HEY1, and C-MYC. Then we downloaded a GEO datasheet of HCC samples (GSE39791) for correlation analyzes. Among those 21 stemness-related genes, S100A4 was positively correlated with OPN, HIF1α, BMI1, CK19, NOTCH1, KLF4, CD44, CD90, TCL, HEY1, and C-MYC (Fig. 4a). We further determined the expression levels of these 11 genes (primers are shown in Supplementary Table S1) in Huh7, PLC, and HepG2 cells with S100A4 overexpression (Fig. 4b, c, d), as well as HCC-LM3 and MHCC97-H cells with S100A4 knockdown (Fig. 4e, f). We found that only the expressions of OPN and its downstream genes, HIF1α and BMI1, were correspondent with S100A4 expressions in all these four cell lines. OPN is a key promoter of HCC metastasis and stemness, but the mechanism of how exosomal S100A4 regulates OPN in HCC is unclear.

**Fig. 5:**
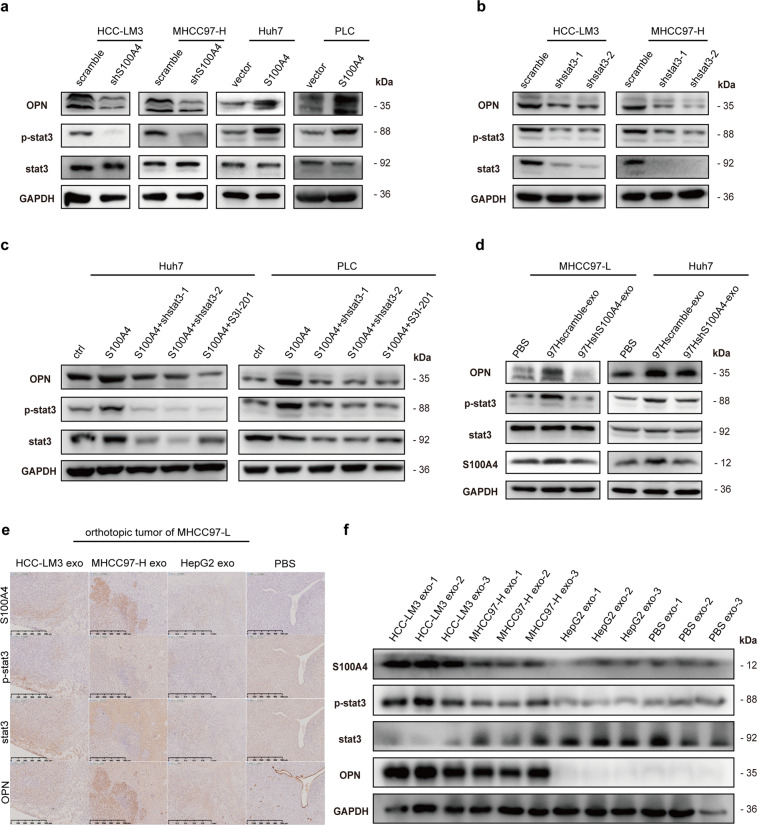
Exosomal S100A4 activates OPN transcription via stat3 phosphorylation. **a** Western blot indicated that OPN and stat3 phosphorylation (p-stat3) levels follow the change of S100A4 level, while stat3 level maintains stable. **b** Western blot showed that knockdown of stat3 expression in high metastatic cell lines (HCC-LM3 and MCC97-H) spontaneously inhibits p-stat3 and OPN expressions. **c** Overexpression of stat3 in low metastatic cell lines (Huh7 and PLC) enhanced p-stat3 and OPN level, while such effect alleviated by stat3 knock down or stat3 inhibitor S3I-201, suggesting a relative up-stream regulation of p-stat3 to OPN. **d** Western blot showed that stat3 phosphorylation and OPN are significantly enhanced after S100A4^rich^ exosomes (97Hshscramble-exo) treatment. **e**, **f** Immunohistochemical staining and Western blot quantification of orthotopic tumors from mice showed that HMH exosomes (HCC-LM3 exosomes and MHCC97-H exosomes) promote stat3 phosphorylation and OPN expression in tumor tissue compared to PBS and LMH exosomes (HepG2 exosomes). Western blot was conducted three times

Interestingly, it was reported that S100A4 could enhance STAT3 phosphorylation,^18,19^ and STAT3 phosphorylation (p-stat3) was correlated with OPN expression.^20^ In addition, STAT3 was predicted to be a potential transcription factor of OPN.^21^ Therefore, we further evaluated OPN, STAT3, and STAT3 phosphorylation levels in S100A4 knockdown HCC-LM3 and MHCC97-H cells and in S100A4 overexpressed Huh7 and PLC cells, and found that OPN and STAT3 phosphorylation levels significantly changed in accordance with S100A4 levels (Fig. 5a). In addition, we knocked down STAT3 in HCC-LM3 and MHCC97-H cells, and observed that STAT3 phosphorylation and OPN expression were both inhibited (Fig. 5b). In Huh7 and PLC cells, when S100A4 was overexpressed, STAT3 phosphorylation and OPN expression were both enhanced, which could be inhibited by stat3 knockdown or stat3 inhibitor, S3I-201 (Fig. 5c). These indicate S100A4 could activate STAT3 phosphorylation and increase OPN expression.

**Fig. 6:**
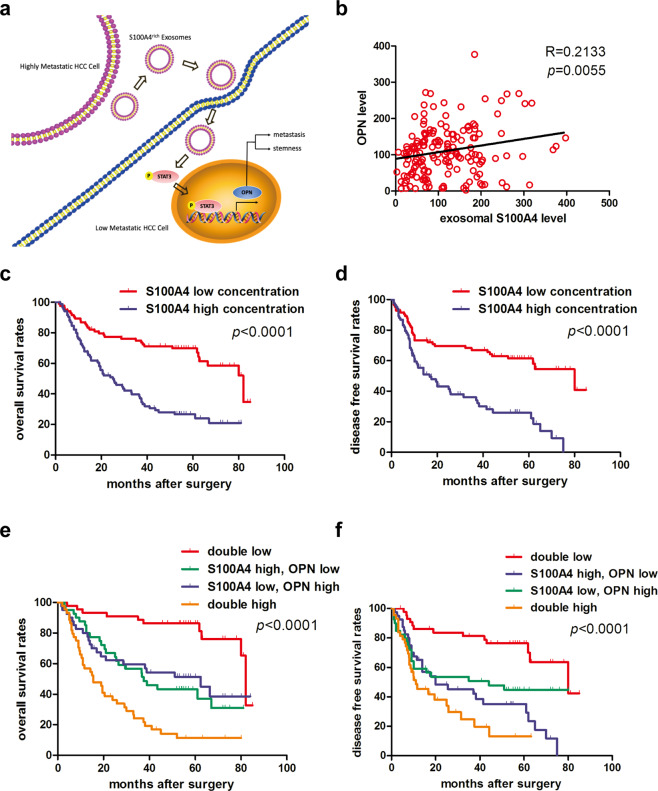
Plasma exosomal S100A4 and OPN levels jointly serve as a powerful postoperative prognostic factor for HCC patients. **a** Working model of exosomal S100A4 promoting HCC metastasis. S100A4^rich^ exosomes released by highly metastatic HCC cells enhanced metastatic potential of low metastatic HCC cells via STAT3 phosphorylation and OPN up-regulation. **b** Correlation analysis on plasma OPN level and plasma exosomal level of S100A4 showed positive correlation in clinical samples. **c**, **d** Patients with low plasma exosomal S100A4 level had significantly better overall survival (OS) and time to treat (TTR) compared with HCC patients with high plasma exosomal S100A4 level. **e**, **f** Patients with both low plasma exosomal S100A4 level and OPN level had the longest OS and DFS among the four subgroups, which were divided according to combination of exosomal S100A4 and OPN. For each cohort, different subgroups were plotted according to the cut-off values of exosomal S100A4 (98.54 pg/ml) and OPN (103.78 ng/ml). Error bars represent the mean ± SD, and the dots represent the value of each experiment; *P < 0.05, **P < 0.01, ***P < 0.001, ****P < 0.0001, ns: no significance. Simple linear regression was employed in (**b**), log-rank test was employed in (**c**–**f**)

To further determine the influence of S100A4^rich^ exosomes on STAT3 phosphorylation and OPN expression, we pre-treated MHCC97-L and Huh7 cells with S100A4^rich^ exosomes before cell lysis and found that S100A4^rich^ exosomes significantly promoted STAT3 phosphorylation and OPN expression, as well as S100A4 expression (Fig. 5d). In addition, when OPN was knocked down or the cells were treated by the STAT3 inhibitor S3I-201, the abilities of invasion and migration of HepG2 and PLC cells were significantly inhibited, even after S100A4^rich^ exosome treatment (Supplementary Fig. S6a–d). Similarly, the sphere formation of HepG2 (Supplementary Fig. S6e) and PLC (Supplementary Fig. S6f) decreased when OPN was knocked down or p-stat3 inhibitor was added, even after with S100A4^rich^ exosomes treatment. Moreover, in orthotopic implantation xenograft tumor of MHCC97-L, HMH-exosomes (HCC-LM3 exosomes and MHCC97-H exosomes) significantly enhanced nuclear STAT3 phosphorylation and cytoplasmic OPN expression while LMH exosomes (HepG2 exosomes) or PBS could not (Fig. 5e). Similar results were also observed with Western blot (Fig. 5f). These results support that S100A4^rich^ exosomes from HMH promote the metastatic potential of LMH, and exosomal S100A4 is a key enhancer for HCC metastasis, by activating STAT3 phosphorylation and up-regulating OPN expression (Fig. 6a).

### Plasma exosomal S100A4 and OPN levels jointly serve as a powerful postoperative prognostic factor for HCC patients

The clinical significance of plasma exosomal S100A4 and OPN levels was further investigated in 168 patients who received curative liver resection for HCC. We first detected the plasma levels of exosomal S100A4, CD63 and CD9 for feasibility verification by Western blot in 4 HCC patients (Supplementary Fig. S7a). We also tested plasma exosomal S100A4 in 10 HCC patients and 10 healthy donors. The exosomal S100A4 was significantly higher in HCC patients (Supplementary Fig. S7b). Exosomal S100A4 was significantly higher in metastatic HCC patients than non-metastatic ones (Supplementary Fig. S7c). Then we determined the plasma exosomal S100A4 and OPN levels by ELISA assay, and found a significantly positive correlation between the exosomal S100A4 and OPN levels (Fig. 6b). Based on the median value of exosomal S100A4 (98.54 pg/ml) and OPN level (103.78 ng/ml), these patients were divided into high- (*n* = 84) and low-exosomal S100A4 group (*n* = 84); as well as high- (*n* = 84) and low-OPN group (*n* = 84). Crosstab analyses showed that exosomal S100A4 level was significantly correlated with serum alpha-fetoprotein (AFP) level, tumor size, vascular invasion, and TNM stage, but not with BCLC stage (Table 1). Plasma OPN level was significantly correlated with vascular invasion, tumor differentiation, and TNM stage, but not with tumor size, number, or BCLC tumor stage (Supplementary Table S2). Furthermore, the 5-year OS and TTR of HCC patients in the low-exosomal S100A4 group were 69.81% and 61.50%, respectively, which were much higher than those in the high-exosomal S100A4 group (26.67% and 26.05%, respectively; *p* < 0.001) (Fig. 6c, d). Based on the combination of exosomal S100A4 level and OPN level, patients were divided into four groups. Among which, HCC patients with low S100A4 and low OPN level had the best prognosis (the longest OS and TTR), and the worst survival was found in those with high S100A4 and high OPN levels (*p* < 0.001) (Fig. 6e, f).

**Table 1:** Relationship between plasma exosomal S100A4 level and clinicopathologic features

Variable	Plasma exosomal S100A4 level (pg/ml)					
	High (*n* = 84)		Low (*n* = 84)		*p*	
	No. of patients	%	No. of patients	%		
Gender					0.170	
Female	14	16.7	8	9.5		
Male	70	83.3	76	90.5		
Age (years)					0.201	
≤50	35	41.7	27	32.1		
50	49	58.3	57	67.9		
HBsAg					0.670	
Negative	14	16.7	12	14.3		
Positive	70	83.3	72	85.7		
HBcAb					0.755	
Negative	6	7.1	5	6.0		
Positive	78	92.9	79	94.0		
Cirrhosis					0.088	
No	13	15.5	6	7.1		
Yes	71	84.5	78	92.9		
ALT (U/L)					0.828	
≤75	72	85.7	71	84.5		
>75	12	14.3	13	15.5		
AFP (ng/mL)					**<0.001**	
≤20	15	17.9	36	42.9		
>20	69	82.1	48	57.1		
Tumor size (cm)					**0.005**	
≤5	30	35.7	48	57.1		
>5	54	64.3	36	42.9		
Tumor number					0.294	
Single	68	81.0	73	86.9		
Multiple	16	9.0	11	13.1		
Tumor capsule					0.436	
None	50	59.5	45	53.6		
Complete	34	40.5	39	46.4		
Tumor thrombus					**0.029**	
No	41	48.8	55	65.5		
Yes	43	51.2	29	34.5		
Tumor differentiation					0.401	
I+II	56	66.7	61	72.6		
III+IV	28	33.3	23	27.4		
TNM stage					**0.045**	
I	34	40.5	47	56.0		
II+III	50	59.5	37	44.0		
BCLC stage					0.087	
0+A	62	73.8	71	84.5		
B+C	22	26.2	13	15.5		

*HBsAg* hepatitis B surface antigen, *HBcAb* hepatitis B core antibody, *AFP* alpha-fetoprotein, *ALT* alanine aminotransferase, *TNM* tumor-node-metastasis, *BCLC* Barcelona Clinic Liver Cancer

Statistical analysis: Chi-Square

Bold values: A *p*-value of 0.05 or lower is considered significant

Univariate analysis showed that exosomal S100A4 level, OPN level, tumor size, tumor capsule, and vascular invasion were significantly associated with OS and TTR of HCC patients (Table 2). Multivariate analysis showed that all of them were independent prognostic indicators for OS, and exosomal S100A4 level, OPN level, tumor size, tumor capsule, and tumor thrombus were independent predictors for TTR (Table 2). The combination of exosomal S100A4 and OPN levels had a better prognostic performance than exosomal S100A4 or OPN alone (Table 2).

**Table 2:** Univariate and multivariate analysis of factors associated with survival and recurrence

	Overall survival		Recurrence free survival	
	HR (95%CI)	*p*	HR (95%CI)	*p*
Univariate analysis				
S100A4^a^ (high vs. low)	**3.056 (1.965-4.752)**	**<0.001**	**2.746 (1.772-4.257)**	**<0.001**
OPN (high vs. low)	**2.556 (1.671-3.910)**	**<0.001**	**1.775 (1.173-2.685)**	**0.007**
Sex (male vs. female)	1.179 (0.627-2.215)	0.609	0.980 (0.545-1.764)	0.947
Age (>50 vs. <50 years)	0.793 (0.522-1.205)	0.278	0.844 (0.554-1.287)	0.431
HBsAg (positive vs. negative)	0.872 (0.501-1.516)	0.627	0.845 (0.486-1.470)	0.551
HBcAb (positive vs. negative)	1.030 (0.450-2.358)	0.944	0.896 (0.391-2.055)	0.796
Cirrhosis (yes vs. no)	0.933 (0.496-1.754)	0.830	1.172 (0.623-2.203)	0.623
ALT (>75 vs. <75 U/L)	0.798 (0.451-1.410)	0.437	1.073 (0.584-1.973)	0.819
AFP (>20 vs. <20 ng/ml)	1.558 (0.965-2.519)	0.070	1.504 (0.941-2.404)	0.088
Tumor size (>5 vs. <5 cm)	**2.431 (1.577-3.749)**	**<0.001**	**1.920 (1.258-2.928)**	**0.002**
Tumor number (multiple vs. single)	1.281 (0.773-2.122)	0.337	0.989 (0.568-1.721)	0.969
Tumor capsule (none vs. complete)	**1.929 (1.249-2.979)**	**0.003**	**1.796 (1.168-2.762)**	**0.008**
Tumor thrombus (yes vs. no)	**2.511 (1.661-3.795)**	**<0.001**	**2.228 (1.476-3.365)**	**<0.001**
Tumor differentiation^b^ (III-IV vs. I-II)	1.222 (0.787 -1.896)	0.372	1.529 (0.988-2.367)	0.057
Combination of S100A4 and OPN				
Double high vs. double low	**9.490 (4.488-20.065)**	**<0.001**	**5.897 (2.874-12.102)**	**<0.001**
Double high vs. (S100A4 high, OPN low)	**2.280 (1.360-3.825)**	**0.002**	1.615 (0.941-2.772)	0.082
Double high vs. (S100A4 low, OPN high)	**2.471 (1.435-4.254)**	**0.001**	**1.812 (1.014-3.239)**	**0.045**
Multivariate analysis^1^				
S100A4 (high vs. low)	**2.554 (1.631-3.999)**	**<0.001**	**2.360 (1.502-3.708)**	**<0.001**
OPN (high vs. low)	**2.423 (1.576-3.724)**	**<0.001**	**1.760 (1.153-2.685)**	**0.009**
Tumor size (>5 vs. <5 cm)	**1.730 (1.098-2.727)**	**0.018**	1.345 (0.859-2.107)	0.195
Tumor capsule (complete vs. none)	**1.740 (1.121-2.703)**	**0.014**	**1.565 (1.016-2.411)**	**0.042**
Tumor thrombus (yes vs. no)	**1.743 (1.132-2.685)**	**0.012**	**1.766 (1.144-2.727)**	**0.010**
Multivariate analysis^2^				
Combination of S100A4 and OPN				
Double high vs. double low	**7.697 (3.561-16.641)**	**<0.001**	**4.795 (2.236-10.281)**	**<0.001**
Double high vs. (S100A4 high, OPN low)	**2.265 (1.343-3.821)**	**0.002**	1.692 (0.978-2.928)	0.060
Double high vs. (S100A4 low, OPN high)	**2.150 (1.233-3.750)**	**0.007**	1.489 (0.819-2.706)	0.192

*AFP* alpha-fetoprotein, *ALT* alanine aminotransferase, *HBsAg* hepatitis B surface antigen, *HBcAb* hepatitis B core antibody, *HR* hazard ratio, *CI* confidence interval

^a^ exosomal S100A4

^b^ Edmondson grade

^1^ Multivariate analysis of S100A4, OPN, Tumor size, Tumor capsule, Tumor thrombus

^2^ Multivariate analysis of Combination S100A4 and OPN, Tumor size, Tumor capsule, Tumor thrombus

Bold values: A *p*-value of 0.05 or lower is considered significant

## Discussion

Metastasis, causing about 90% deaths of cancer patients, is well known as the most significant characteristic of malignant cancer.^1,2^ Intratumor heterogeneity, which fosters tumor evolution, is a key challenge for cancer treatment.^22^ Exosomes have been reported to mediate intercellular communication and serve as a powerful promotor to cancer invasiveness.^5^ Exosomes are derived from multivesicular bodies, which are formed by almost all types of cells.^6^ Tumor cells might release more exosomes than normal cells, the amount of which might be positively correlated with malignancy.^9^ In our study, based on intra-tumor heterogeneity theory, we utilized HCC cell lines with different metastatic potentials for functional and mechanistic study of exosomes related HCC metastasis.

Previously published literature reported that exosomes could transfer mutated EGFRvIII protein to promote malignancy of EGFRvIII wildtype glioma,^8^ and HCC-derived exosomes could mobilize normal hepatocyte.^23^ We observed that HMH-exosomes could significantly enhance metastatic potential of MHCC97-L and HepG2, both low metastatic HCC cells. This phenomenon was also observed by Wang et al. that exosomal circPTGR1 from LM3 cells could increase migratory and invasive ability of HepG2 cells.^24^ In order to determine the key functional factor in HMH-exosomes, we adopted iTRAQ spectrum mass for screening, and found that members of 4 protein families were significantly up-regulated in HMH-exosomes, including Apo, PSMA/B, EEF/EIF, and S100 calcium binding protein family. Recent papers have demonstrated that S100A protein family played key roles in cancer metastasis and pre-metastatic niche formation.^25^ In addition, S100A4 might be vital for cancer stemness and metastasis.^15,26–28^ Consistently, our data showed that S100A4 expression in HCC cell lines and cell line derived exosomes were positively correlated with metastatic potential of these cell lines. S100A4 expression had a great influence on migration and invasion abilities of HCC cells, as well as sphere formation and tumor initiation assays. Furthermore, we obtained S100A4^low^ and S100A4^rich^ exosomes from S100A4 knockdown MHCC97-H cells and controlled counterparts, based on research reported by Peinado H et al.^9^ As expected, we observed that migration and invasion in vitro along with lung metastatic potential in vivo of MHCC97-L were significantly enhanced by S100A4^rich^ exosomes, but not S100A4^low^ exosomes. Similar results could be observed in sphere formation and tumor initiation assays.

We further selected 21 stemness-related genes based on 3 published studies^29–31^ and analyzed their relationship with S100A4 expression in HCC tissues (GSE39791). Ultimately, we focused on OPN, a typical HCC promotor^32,33^ significantly upregulated by S100A4. Extracellular S100A4 was reported to upregulate OPN via NF-κB pathway in osteosarcoma,^34^ and to maintain STAT3 phosphorylation level in neuron for functional protection of the brain.^18^ Coincidently, Yang XL et al. reported that S100A4 could promote STAT3 phosphorylation in HCC.^15^ Combined with another literature indicating that STAT3 phosphorylation might induce OPN expression in HCC,^35^ and with our experiment data, we proposed that S100A4^rich^ exosomes could up-regulate OPN expression in low metastatic HCC cells via STAT3 phosphorylation. Our findings suggested that exosomal S100A4 induce the expression of OPN via stat3 phosphorylation but not NF-κB- signaling. This novel finding adds more information to how S100A4 mediates the metastatic progression. Interestingly, Jiao et al. reported that S100A4 + stromal cells could maintain HCC stemness, which suggests a crosstalk between inflammation and stemness.^36^ Our study suggested exosomal S100A4 could play a role in S100A4 + cell subpopulation in the crosstalk with cancer stem cells.

Mortality of HCC mostly results from high recurrence rate even after curative surgical resection.^37^ The predictive biomarkers for recurrence of HCC have great benefit for clinical decision making and follow-up procedure establishment. Recently studies showed great potential of exosomes as predictive biomarker. Wang et al. found that miR-21 is enriched in serum exosomes of HCC patients which may serve as a diagnostic biomarker.^38^ Decreased expression of serum exosomal miR-718 was associated with recurrent HCC.^39^ In this study, we found that plasma exosomal S100A4 and plasma OPN levels were significantly associated with prognosis of HCC patients, and the combination of exosomal S100A4 and OPN had a better prognostic performance than each alone.

In summary, our findings demonstrated a vital role of exosomal S100A4 in regulating stemness and metastatic potential of HCC cells. Exosomal S100A4 released by highly metastatic HCC cells enhanced metastatic potential of low metastatic HCC cells via STAT3 phosphorylation and OPN up-regulation. Moreover, plasma exosomal S100A4 level combined with plasma OPN level was determined as a powerful prognostic predictor for postoperative HCC patients. Our study highlights a novel function of exosomal S100A4 in regulating HCC stemness and provides an insight into the participation of S100A4 in exosome-mediated communication in HCC.

## Materials and methods

### Cell lines

HCC cell lines HCC-LM3, MHCC97-H, and MHCC97-L were established at the Liver Cancer Institute, Fudan University. They have genetically identical backgrounds and stepwise increasing metastatic potentials.^40^ The Huh7, PLC/PRF/5 (PLC), and HepG2 cell lines were purchased from the Shanghai cell bank, Chinese Academy of Sciences. All cell lines were cultured in Dulbecco’s modified Eagle’s medium (DMEM, Hyclone, Logan, UT, USA) supplemented with 10% fetal bovine serum (FBS, Gibco, Carlsbad, CA, USA) and maintained in a humidified incubator with 5% CO_2_ at 37 °C.

### Exosomes isolation

Concerning cells were cultured in DMEM with 10% FBS until they reached 80% confluence. The cell culture medium was removed. The cells were washed once with PBS and then cultured in cell culture medium containing no serum for 48 h. Cell culture medium was centrifuged at 3000 g for 15 min to eliminate cell debris. The exosomes were extracted by EXOTC50A-1 (System Biosciences, Palo Alto, CA, USA) according to manufacturer’s instructions. Exosomes were isolated from plasma specimen by EXOQ5TM-1 (System Biosciences), according to the manufacturer’s instructions.

Briefly, 5 mL of centrifuged supernatant was mixed with 1 mL of ExoQuick-TC/TM solution by inverting the tube several times. The sample was incubated overnight at 4 °C then centrifuged twice at 1500 g for 30 and 5 min, respectively, in order to remove the supernatant. The pellet was re-suspended in sterilized PBS, quantified by bicinchoninic acid (BCA) assays (Thermo Fisher Scientific, Waltham, MA, USA).

### In vitro sphere formation, migration, and invasion assays

Sphere formation was performed by plating 1000 cells per well into 6-well ultra-low attachment plate (Corning Incorporated Life Sciences, NY, USA) in serum-free DMEM/F12 medium (Gibco), supplemented with B27 (1:50; Invitrogen, Thermo Fisher Scientific, Waltham, MA, USA), N2 (1:100; Invitrogen), 20 ng/ml bFGF, 10 ng/ml EGF (Peprotech, Rocky Hill, NJ, USA) and 20 μg/ml exosomes or 100 μl PBS as control. Cells were incubated in a 5% CO_2_ incubator at 37 °C for 1 week. For passaging of primary spheres to secondary spheres, 0.25% trypsin (Gibco) was used, and 1000 cells were re-seeded into 6-well ultra-low attachment plate for additional one week. The number of tumor spheres per-well was counted under an inverted microscope (×100 or × 40, Olympus Corporation, Tokyo, Japan).

The migration and invasion ability of HCC cells were determined by using 24-well Transwell chambers, with upper and lower culture compartments separated by polycarbonate membranes with 8 µm pores (BD Biosciences, Franklin Lakes, NJ, USA). The bottom chamber was filled with DMEM containing 10% FBS as a chemoattractant. HCC cells were co-cultured with 20 μg/ml exosomes for 24 h, PBS as control. Cells, 5 × 10^4^ cells for migration and 10 × 10^4^ cells for invasion, in serum-free medium were seeded into the upper chamber and maintained at 37 °C in a humidified incubator containing 5% CO_2_. Cells that migrated to the underside of the membrane were stained with crystal violet, imaged, and counted with light microscope (×100, Leica, Wetzlar, Germany).

All the in vitro assays were conducted three times with three repetitions.

### Establishment of in vivo tumor models

For the assessment of tumor initiation abilities, 1000 cells were suspended in 100 μl of PBS (Hyclone) and Matrigel (BD Biosciences) mix (1:1) and implanted subcutaneously into the armpit of 4- to 6-week-old NOD/SCID female mice. Tumor formation was monitored weekly.

For orthotopic implantation xenograft models, xenografts were established by subcutaneously implanting 5 × 10^6^ cells into male nude mice (BALB/c nu/nu) that were 4–6 weeks old. Then subcutaneous tumors were removed and dissected into 1 mm^3^ sections, and then were planted into the liver of nude mice to establish orthotopic implantation tumor models. For different groups, 20 μg exosomes or 100 μl PBS per mouse were intravenously injected through the tail vein twice a week. PBS was used as control. Exosomes were quantified by BCA assays. Mice were sacrificed after 6 weeks. Tumors, livers, and lungs were removed, fixed in formalin, and embedded in paraffin. Consecutive sections were made for each lung tissue block and stained with hematoxylin and eosin. The number of lung metastasis was calculated and evaluated independently by two pathologists.

For the experimental lung metastasis model, target cells were co-cultured with 20 μg/ml exosomes for 24 h. Then, we injected 5 × 10^4^ cells along with 20 μg exosomes or 100 μl PBS as control, into tail veins of nude mice. Exosomes were intravenously injected through the tail vein twice a week with 20 μg exosomes per mouse or 100 μl PBS as control. Mice were sacrificed 4 weeks after HCC cell injection for further lung fixed with paraffin embedding. Consecutive sections were made for each lung tissue block and stained with hematoxylin and eosin. The number of lung metastasis was calculated and evaluated independently by two pathologists.

All experimental procedures involving animals were approved by The Animal Care and Use Committee of Shanghai Medical College, Fudan University, China.

## Supplementary information

Supplementary_Materials

## Data Availability

The datasets used and analyzed during the current study are available from the corresponding author on reasonable request. Additional methods were described in the Supplementary Information.
