# Supplementary material for: Exosomal S100A4 derived from highly metastatic hepatocellular carcinoma cells promotes metastasis by activating STAT3
Source: Signal Transduct Target Ther. 2021 May 26;6:187. doi: 10.1038/s41392-021-00579-3 (PMC8149717; doi:10.1038/s41392-021-00579-3)
Supplement: Supplementary file 1 — Supplementary_Materials [file 41392_2021_579_MOESM1_ESM.docx]

Supplementary Materials for

Exosomal S100A4 Derived from Highly Metastatic Hepatocellular Carcinoma Cells Promotes Metastasis by Activating STAT3

Authors: Haoting Sun, Chaoqun Wang, Beiyuan Hu, Xiaomei Gao, Tiantian Zou, Qin Luo, Mo Chen, Yan Fu, Yuanyuan Sheng, Kaili Zhang, Yan Zheng, Xudong Ren, Shican Yan, Yan Geng, Luyu Yang, Qiongzhu Dong^*^, Lunxiu Qin^*^

Correspondence to: [qinlx@fudan.edu.cn](mailto:qinlx@fudan.edu.cn) and qzhdong@fudan.edu.cn

**This PDF file includes:**

Materials and Methods

Abbreviations

Figures. S1 to S7

Tables S1 to S2

**Other Supplementary Materials for this manuscript include the following:**

None

Materials and Methods

Patients, follow-up, and clinical specimens

A total of 168 patients who received curative resection for HCC at authors’ institutes from January 2010 to December 2012 were enrolled in this study. None of them received any preoperative cancer treatment. The clinical samples were collected from patients with written informed consent in accordance with ethical approval by the Ethics Committee of Huashan Hospital, Fudan University (Shanghai, China). Plasma samples were collected before surgery and stored at -80°C until further processing.

The patients were followed-up after surgical treatment until April 2018, with a median follow-up time of 38.7 months (range, 2-85 months). Serum alpha-fetoprotein (AFP) level and liver ultrasonography were monitored every 2 months during the follow-up period. Computed tomography (CT) or magnetic resonance imaging (MRI) scan was performed every 6 months or when recurrence was suspected. All these examinations were performed independently by doctors without knowing this study.

The overall survival (OS) was calculated from the date of operation to the date of death or to the date of last follow-up. The time to recurrence (TTR) was calculated from the date of resection to the date when tumor recurrence was diagnosed, if recurrence was not diagnosed during the period of study, the cases were censored on the date of death or the last date of follow-up.

Analysis of exosomes

Exosomes were characterized by FEI Tecnai G2 Spirit BioTwin transmission electron microscope (FEI Company, USA) with accelerating voltage of 120kV. NanoSight NS300 (Malvern Instruments Ltd, UK) was used for quantification.

Western blot

Detailed methods were described in supporting materials.

Total protein was extracted by lysing cells in RIPA buffer containing protease inhibitor. Protein samples were separated by sodium dodecyl sulfate polyacrylamide gel electrophoresis (SDS-PAGE) and transferred onto poly vinylidene fluoride (PVDF) membranes. After blocking with 5% non-fat milk in TBS-T, membranes were incubated with primary antibody. The following antibodies were used: anti-S100A4 (1:1000, Proteintech, Rosemont, IL, USA), anti-OPN (1:1000, Proteintech), anti-CD63 (1:1000, Proteintech), anti-CD9 (1:1000, Proteintech), anti-stat3 and anti-p-stat3 (1:1000, CST, Danvers, MA, USA), anti-β-actin (1:2000, Abcam), anti-GAPDH (1:2000, CST). Protein bands were detected by using Image Acquisition using ImageQuant™ LAS 4000 (GE Healthcare Life Sciences, Pittsburgh, PA, USA).

RNA isolation, reverse-transcription, and quantitative real-time polymerase chain reaction (qPCR)

RNA of cell lines was isolated using Trizol reagent (Invitrogen). RNA was quantified using a Nanodrop ND-1000 (Thermo Fischer Scientific). Complementary DNA synthesis was performed using PrimeScript reverse transcriptase reagent kit (Takara Bio, Mountain View, CA, USA) according to the manufacturer’s directions.

Real-time PCR was performed using SYBR Green PCR Master Mix (DBI Bioscience, Ludwigshafen, Germany) and ABI PRISM 7900 Sequence Detection System (Applied Biosystems, Thermo Fischer Scientific). Results were normalized to β-actin for mRNA measurement. Fold change was calculated by the 2-ΔΔCt method where ΔΔCt=ΔCt (Target-Reference) Treatment – ΔCt (Target-Reference) control. All the primers were listed in Supporting Table S1. qPCR was conducted three times with three repetitions.

iTRAQ assay

Exosome samples were resuspended approximately 8 times with Lysis buffer (4% SDS, 100 mM Hepes PH=7.6 containing protease inhibitor cocktail and PMSF). The homogenate was sonicated 30 min on ice. After centrifugation at 25 000 g for 30 min at 4℃, the supernatant was collected and stored at -80℃. The total protein concentration was measured using BCA Kit. Proteins were reduced in 50-mM Tris-(2-carboxyethyl) phosphine at 30°C for 1h. Protein samples were then allowed to cool down to room temperature, and the cysteines were blocked with 1μl of 200-mM methyl methane thiosulfonate (MMTS) at room temperature for 10min. Subsequently, proteins were isotopically labeled with iTRAQ reagents (Applied Biosystems, Thermo Fischer Scientific) at room temperature for 1h. The labeling reaction was then stopped by the addition of 10μl of 1-M ethanolamine. Then, the samples were separated and identified with 2D-nano-HPLC (Shimadzu, Kyoto, Japan) and MALDI-TOF-TOF 4700 (Applied Biosystems, Thermo Fischer Scientific) according to Carranza *et al.* (1).

Enzyme linked immunosorbent assay (ELISA)

Plasma exosomal S100A4 and plasma OPN levels in patients with HCC were assessed using the Human S100A4/FSP1 ELISA Kit (LifeSpan BioSciences, Seattle, WA，USA) and Human Osteopontin Platinum ELISA kit (eBioscience, Thermo Fischer Scientific), according to the manufacturer’s instructions.

Vectors and cell transfections

Expression vector mediated by lentivirus for human S100A4 was constructed. The sequence of S100A4 was amplified from cDNA library via specific primers: forward primer-5’-AGCTAGCATGGCGTGCCCTCTGGA-3’, reward primer-5’-CGGATCCTCATTTCTTCCTGGGCTG-3’. Then harvested DNA was inserted into pCDH-puro expression vector (System Biosciences).

ShRNA for S100A4 was constructed depending on serial number, TRCN0000053609, from sigma official website.

Methods of cell transfections were described previously (2).

Statistical analysis

Statistical analyses were performed using the Statistical Package for Social Sciences Version 16.0 (SPSS16.0) and Graphpad Prism® 5.0 software. The χ2 test, Student’s t test and one-way ANOVA were used for comparison between groups. The correlation was determined by Pearson analysis. Kaplan-Meier survival analyses were used to estimate the prognostic value, and the log-rank test was used to assess the survival differences. Univariate and multivariate Cox regression analyses were performed to evaluate differences of all possible factors in the risk of death and recurrence. p<0.05 was considered statistically significant.

**REFERENCE**

1 Carranza, P. et al. A gel-free quantitative proteomics approach to investigate temperature adaptation of the food-borne pathogen Cronobacter turicensis 3032. Proteomics. 10, 3248-3261, (2010).

2 Dong, Q. et al. Osteopontin promotes epithelial-mesenchymal transition of hepatocellular carcinoma through regulating vimentin. Oncotarget. 7, 12997-13012, (2016).

Supplementary Text

Abbreviations**:**

HCC, hepatocellular carcinoma; HMH, highly metastatic HCC cells; LMH, low metastatic HCC cells; iTRAQ, isobaric tags for relative and absolute quantitation, S100A4, S100 calcium-binding protein A4; OPN, osteopontin; OS, overall survival; TTR, time to recurrence; STAT3, signaling transducer and activator of transcription 3; TNM: Tumor-node-metastasis;


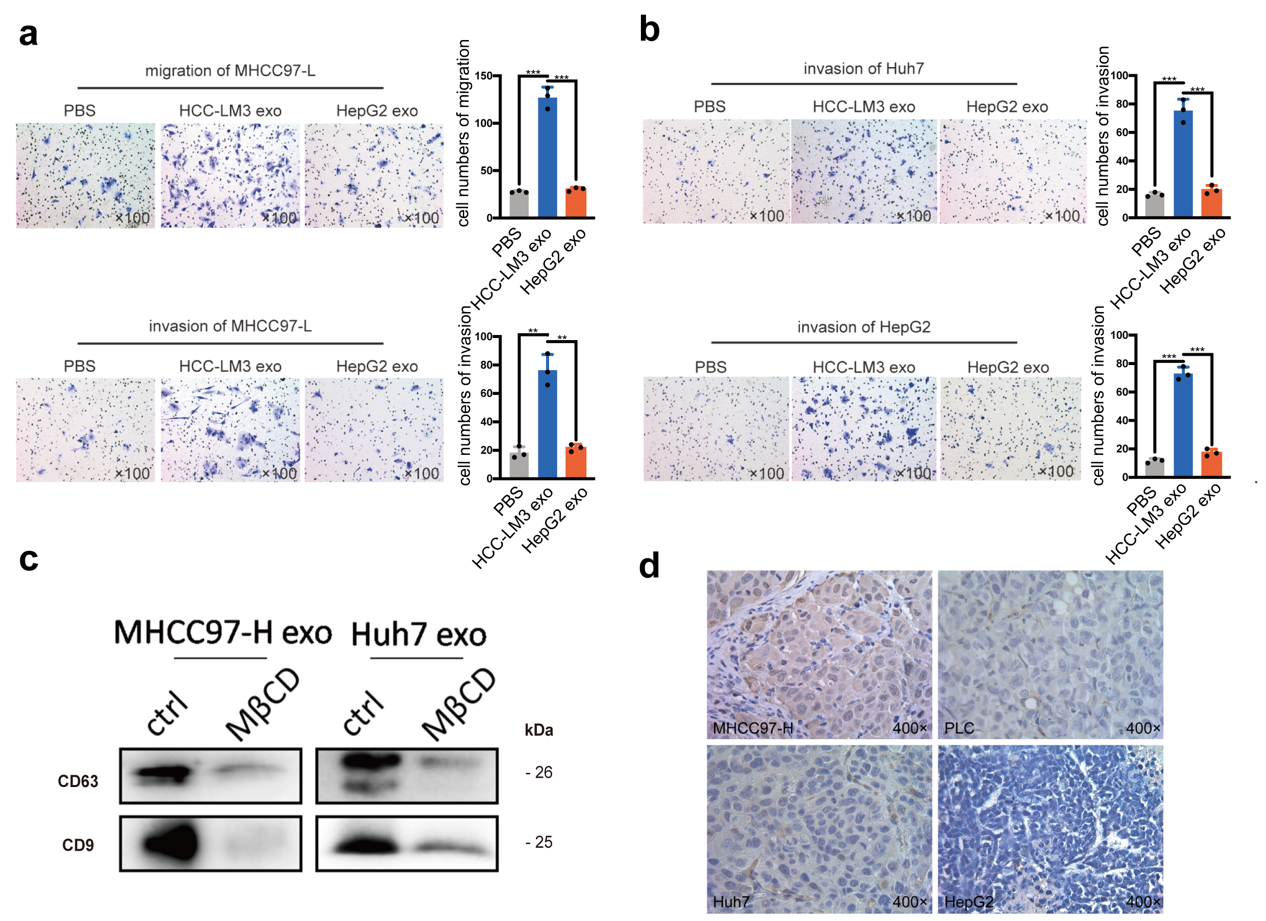


Figure. S1.

Supplementary Fig. S1. (a, b) LHM (MHCC97-L, Huh7 and HepG2) pre-treated with HCC-LM3 exosomes, HepG2 exosomes or PBS (as negative control) for 24 hours. The migration ability of MHCC97-L and invasion ability of all the three cell lines were significantly enhanced by HCC-LM3 exosomes compared to the control groups. All the in vitro assays were conducted three times with three repetitions. (c) Western blot reveals that after MβCD treatment exosomal markers sharply decreased, suggesting the effective isolation of exosomes. (d) Representative immunohistochemistry of S100A4 expression in HCC cell lines derived tumor. S100A4 was highly expressed in MHCC97-H derived tumors compared with tumors derived from low metastatic potential cell lines (PLC, Huh7 and HepG2). All the *in vitro* assays were conducted three times with three repetitions. Error bars represent the mean ± SD, and the dots represent the value of each experiment; *P < 0.05, **P < 0.01, ***P < 0.001, ****P < 0.0001. An unpaired *t* test was employed in (a) and (b).


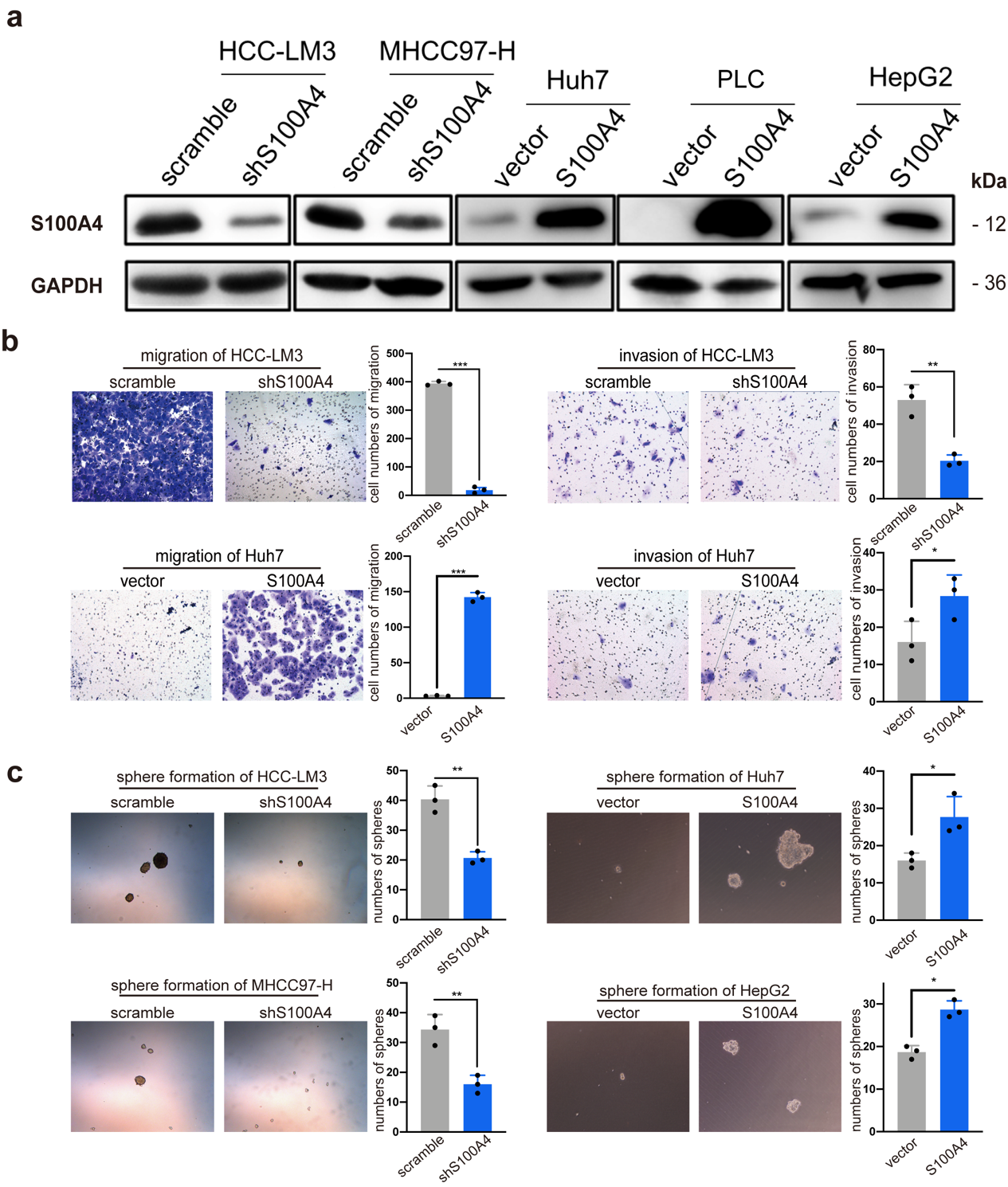


Figure. S2.

Supplementary Fig. S2. (a) Western bolt verified the S100A4 knock-down in HCC-LM3 and MHCC97-H cells, and S100A4 overexpression in PLC, Huh7 and HepG2 cells. (b) S100A4 knock-down in HCC-LM3 results in inhibited migration and invasion ability. Overexpression of S100A4 enhanced migration and invasion in Huh7. (c) Sphere formation assay in vitro revealed that knock-down of S100A4 in HCC-LM3 and MHCC97-H cells resulted in less and smaller sphere formation while overexpression of S100A4 in Huh7 and HepG2 cells promoted sphere formation. All the *in vitro* assays were conducted three times with three repetitions. Error bars represent the mean ± SD, and the dots represent the value of each experiment; *P < 0.05, **P < 0.01, ***P < 0.001, ****P < 0.0001. An unpaired *t* test was employed in (b) and (c).


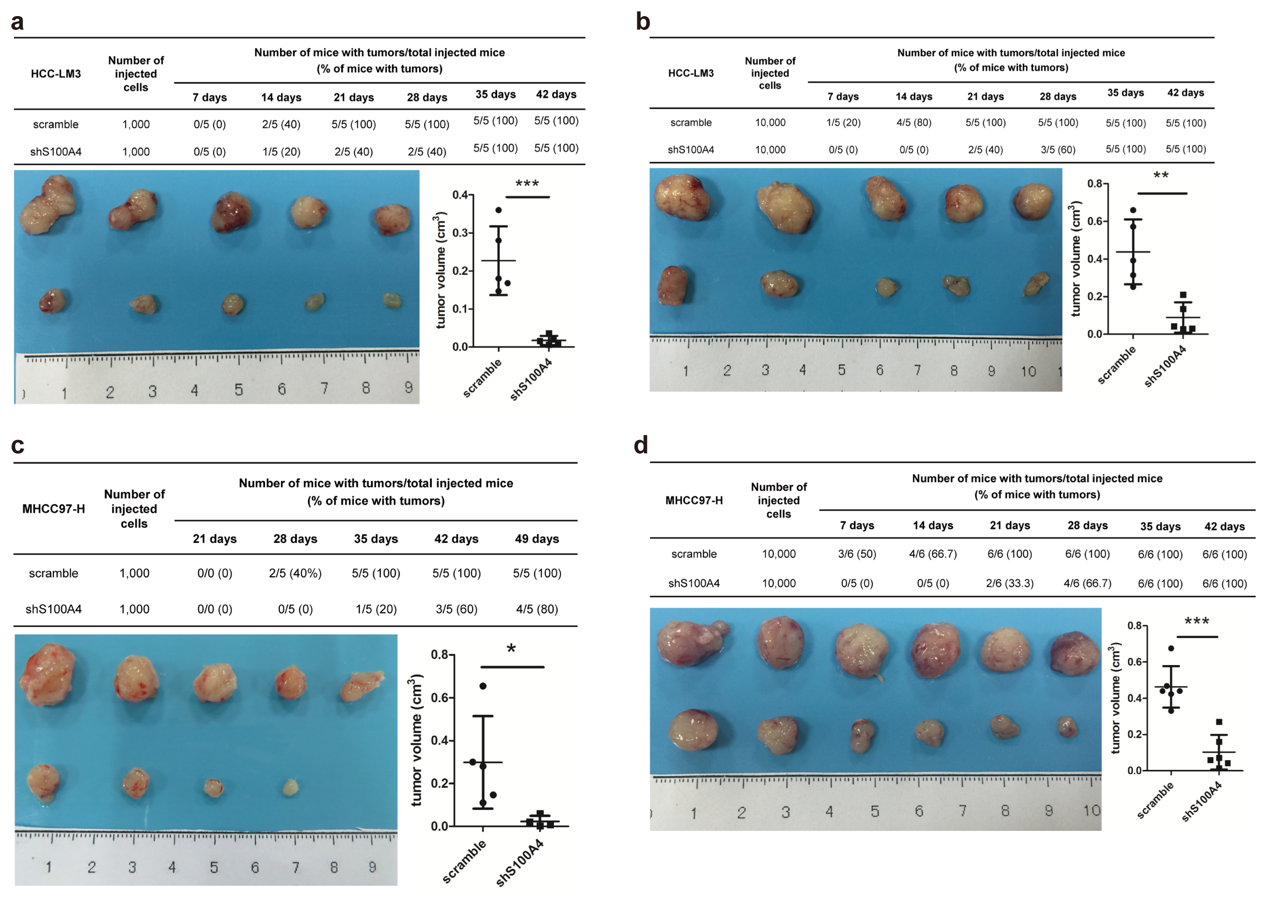


Figure. S3.

Supplementary Fig. S3. *In vivo* tumor initiation experiment with S100A4 knock-down highly metastatic HCC cells (HCC-LM3) showed delayed tumor initiation time and decreased tumor volume. Error bars represent the mean ± SD, and the dots represent the volume of each tumor; *P < 0.05, **P < 0.01, ***P < 0.001, ****P < 0.0001. Statistical significance was determined by unpaired *t* test.


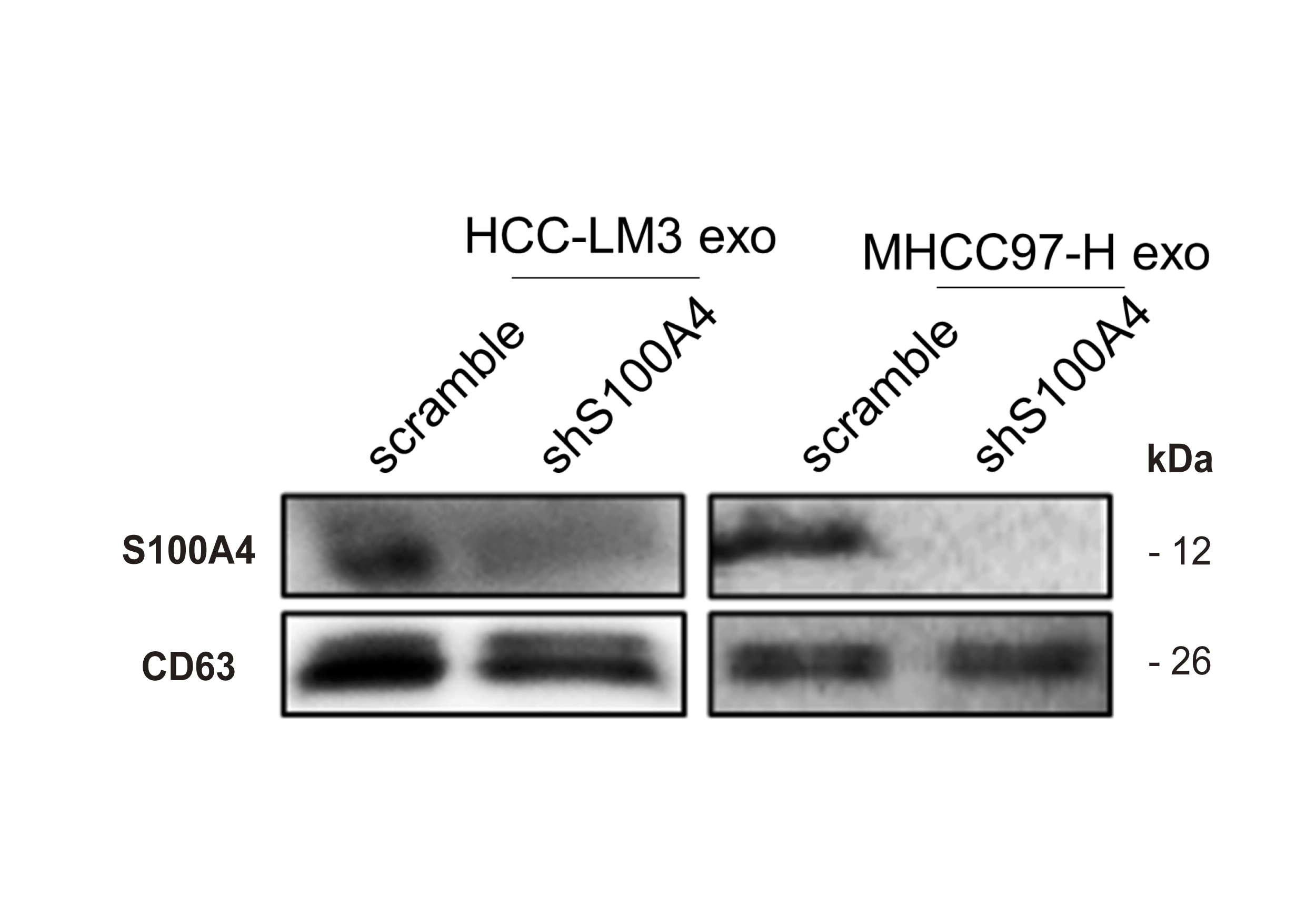


Figure. S4.

Supplementary Fig. S4. Western bolt confirmed the knockdown of S100A4 in exosomes derived from HCC-LM3 and MHCC97-H cells. Exosomes of MHCC97-H cells with S100A4 knockdown were defined as S100A4^low^ exosomes, ones derived from the scrambled counterparts were defined as S100A4^rich^ exosomes.


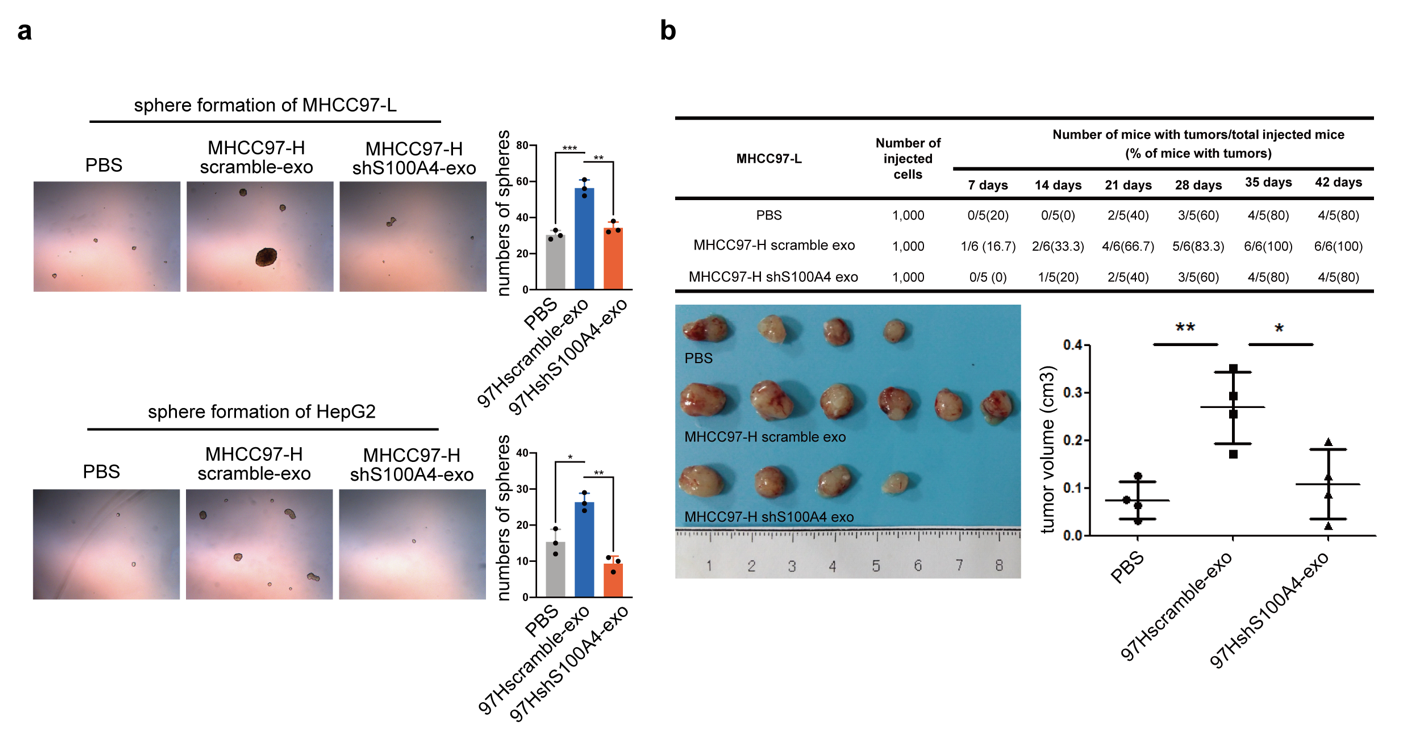


Figure. S5.

Supplementary Fig. S5. (a) Sphere formation assay in vitro confirmed enhanced sphere formation rates of MHCC97-L and HepG2 cells after S100A4-rich exosomes (MHCC97-H scramble-exo) treatment. (b) *In vivo* experiment with mice models injected with MHCC97-L cells treated with S100A4^rich^ exosomes (MHCC97-H scramble-exo) had faster tumor initiation and increased tumor volume compared with control groups (S100A4^low^ or PBS treatment). All the *in vitro* assays were conducted three times with three repetitions. Error bars represent the mean ± SD, and the dots represent the value of each experiment; *P < 0.05, **P < 0.01, ***P < 0.001, ****P < 0.0001. An unpaired t test was employed in (a), and one-way ANOVA followed by Bonferroni’s post hoc test was employed in (b).


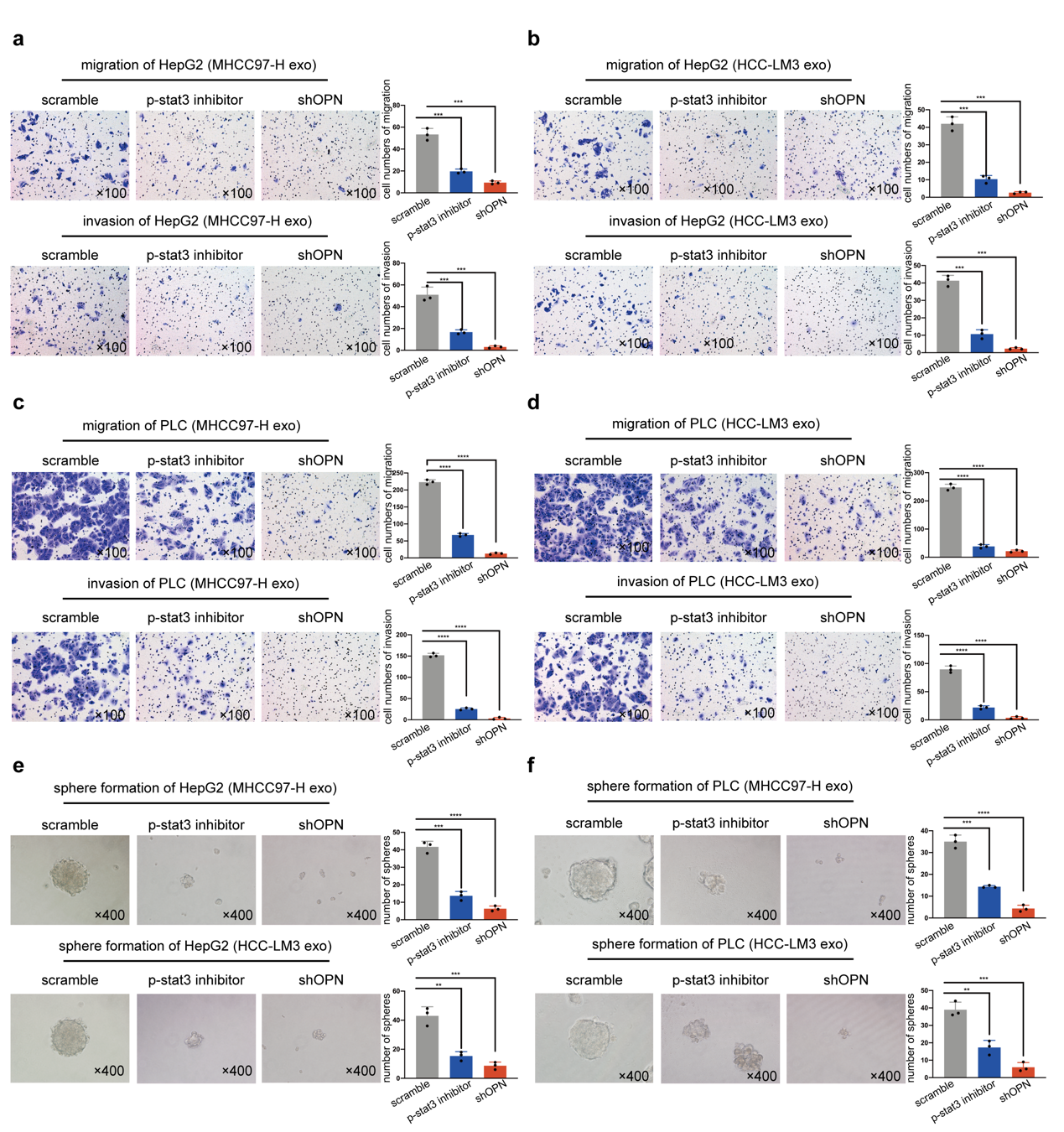


Figure. S6.

Supplementary Fig. S6. Assessment of migration (upper panel) and invasion (lower panel) of HepG2 (a, b) and PLC (c, d) *in vitro*. Low metastatic HCC cells (HepG2 and PLC) were pre-treated with p-stat3 inhibitor for 48h or knocked down OPN then treated with MHCC97-H exo (a, c) and HCC-LM3 exo (b, d) for 24h. Assessment of sphere formation of HepG2 and PLC in vitro. Low metastatic HCC cells of HepG2 (e) and PLC (f) were pre-treated with p-stat3 inhibitor for 48h or knockdown of OPN then treated with MHCC97-H exo (upper panel) and HCC-LM3 exo (lower panel) for 10 days. All the in vitro assays were conducted two times with three repetitions. Error bars represent the mean ± SD, and the dots represent the value of one representative experiment; *P < 0.05, **P < 0.01, ***P < 0.001, ****P < 0.0001. Statistical significance was determined by unpaired *t* test.


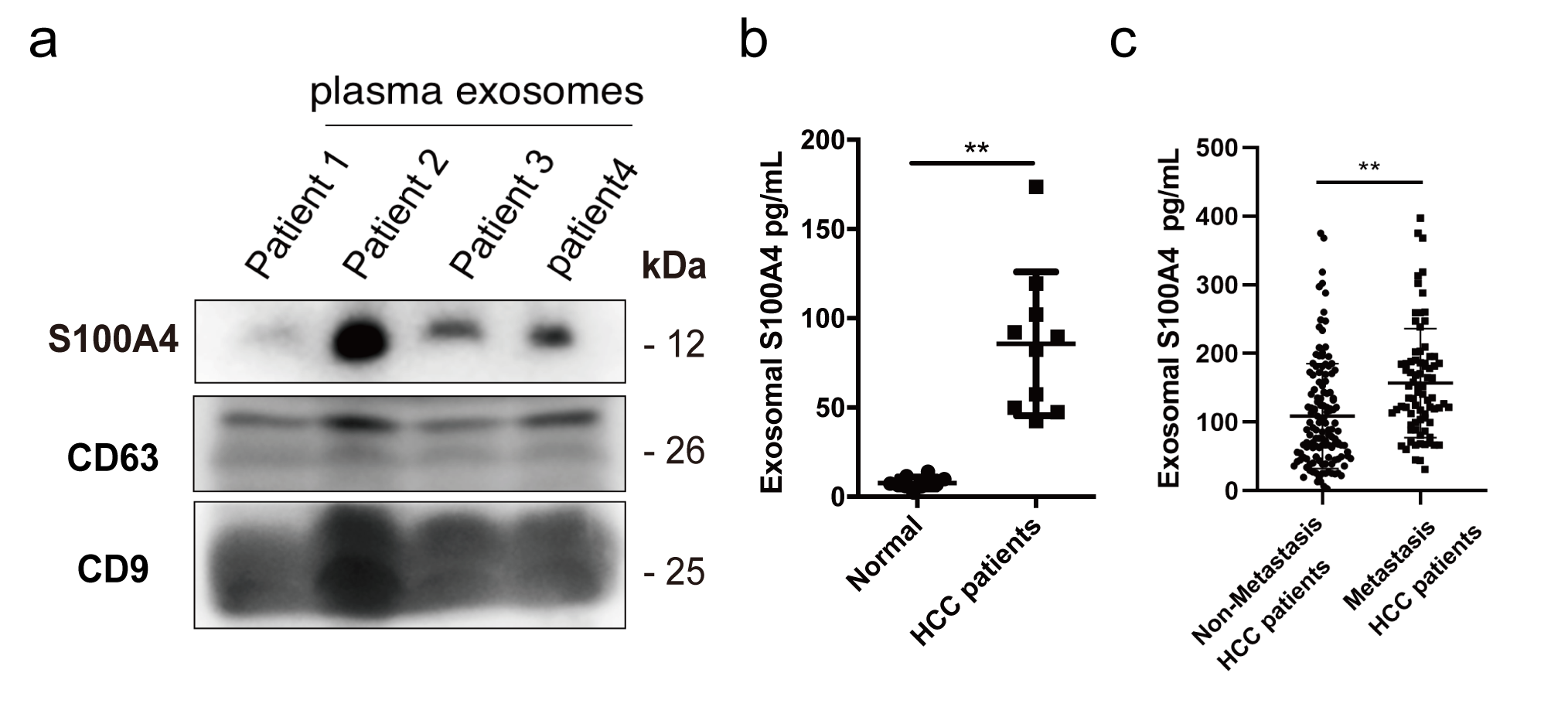


Figure. S7.

Supplementary Fig. S7. (a) Western bolt confirmed the exosomal expression of S100A4, CD63 and CD9 in 4 HCC patients. (b) Plasma exosomal S100A4 were tested in 10 HCC patients and 10 healthy donors by ELISA. (c) Plasma exosomal S100A4 in metastatic and non-metastatic HCC patients. *P < 0.05, **P < 0.01, ***P < 0.001, ****P < 0.0001. Statistical significance was determined by unpaired *t* test.

Table S1. Primer sequences of HCC stemness-related genes

| Primer name | Sense | Anti-sense |
| --- | --- | --- |
| OPN  HIF1α | CTCCATTGACTCGAACGACTC  CTCTCCTCTTCCTTCCTCCA | CAGGTCTGCGAAACTTCTTAGAT  GGTCTTCACCTGTTTGTAGCTG |
| BMI1 | CCACCTGATGTGTGTGCTTTG | TTCAGTAGTGGTCTGGTCTTGT |
| CK19 | ACCAAGTTTGAGACGGAACAG | CCCTCAGCGTACTGATTTCCT |
| NOTCH1 | GAGGCGTGGCAGACTATGC | CTTGTACTCCGTCAGCGTGA |
| KLF4 | CAGCTTCACCTATCCGATCCG | GACTCCCTGCCATAGAGGAGG |
| CD44 | CTGCCGCTTTGCAGGTGTA | CATTGTGGGCAAGGTGCTATT |
| CD90 | ATCGCTCTCCTGCTAACAGTC | CTCGTACTGGATGGGTGAACT |
| TCL | ACTTGCTCGGACTGTATGACA | CCGTGTTGGGGTAGGAGAGT |
| HEY1 | GTTCGGCTCTAGGTTCCATGT | CGTCGGCGCTTCTCAATTATTC |
| C-MYC | GTCAAGAGGCGAACACACAAC | TTGGACGGACAGGATGTATGC |

Table S2. Relationship between plasma OPN level and clinicopathologic features

| Variable | Plasma OPN level (pg/ml) | | | | |
| --- | --- | --- | --- | --- | --- |
|  | High (n=84) | | Low (n=84) | | *p* |
|  | No. of patients | % | No. of patients | % |  |
| Gender  Female  Male  Age (years)  ≤50  >50  HBsAg  Negative  Positive  HBcAb  Negative  Positive  Cirrhosis  No  Yes  ALT (U/L)  ≤75  >75  AFP (ng/mL)  ≤20  >20  Tumor size (cm)  ≤5  >5  Tumor number  Single  Multiple  Tumor capsule  None  Complete  Tumor thrombus  No  Yes  Tumor differentiation  I+II  III+IV  TNM stage  I  II+III  BCLC stage  0+A  B+C | 7  77  30  54  17  67  8  76  12  72  69  15  21  63  33  51  74  10  51  33  41  43  51  33  34  50  64  20 | 8.3  91.7  35.7  64.3  20.2  79.8  9.5  90.5  14.3  85.7  82.1  17.9  25.0  75.0  39.3  60.7  88.1  11.9  60.7  39.3  48.8  51.2  60.7  39.3  40.5  59.5  76.2  23.8 | 15  69  32  52  9  75  3  81  7  77  74  10  30  54  45  39  67  17  44  40  55  29  66  18  47  37  69  15 | 17.9  82.1  38.1  61.9  10.7  89.3  3.6  96.4  8.3  91.7  88.1  11.9  35.7  64.3  53.6  46.4  79.8  20.2  52.4  47.6  65.5  34.5  78.6  21.4  56.0  44.0  82.1  17.9 | 0.067  0.749  0.088  0.211  0.223  0.828  0.131  0.063  0141  0.276  **0.029**  **0.012**  **0.045**  0.342 |

Abbreviations: HBsAg, hepatitis B surface antigen; HBcAb, hepatitis B core antibody; AFP, alpha-fetoprotein; ALT, alanine aminotransferase; TNM, tumor-node-metastasis; BCLC, Barcelona Clinic Liver Cancer

Statistical analysis: Chi-Square and Fishers Exact Test
